
JOURNAL INFORMATION
==============================
NLM Title Abbreviation: Physiol Rep
Journal ID: Physiol Rep
NLM Journal ID: 101607800
Journal ID: PHY2

Physiological Reports

EISSN: 2051-817X
Publisher: Wiley

ARTICLE INFORMATION
==============================
PMCID: PMC11474657
DOI: 10.14814/phy2.15328
Article ID: PHY215328
Article version: 1
Subjects: Issue Information, Issue Information

Issue Information

Electronic publication date: 2023 Sep 28
Publication date: 2023 Oct
Volume: 11
Issue: 19
Electronic Location ID: e15328
Copyright: © 2023 The Authors. Physiological Reports published by Wiley Periodicals LLC on behalf of The Physiological Society and the American Physiological Society.
License: This is an open access article under the terms of the http://creativecommons.org/licenses/by/4.0/ License, which permits use, distribution and reproduction in any medium, provided the original work is properly cited.
License URL: https://creativecommons.org/licenses/by/4.0/

Figure count: 0
Table count: 0
Page count: 3

source-schema-version-number: 2.0
cover-date: October 2023
details-of-publishers-convertor: Converter:WILEY_ML3GV2_TO_JATSPMC version:6.4.9 mode:remove_FC converted:15.10.2024

==============================

### PHYSIOLOGICAL REPORTS

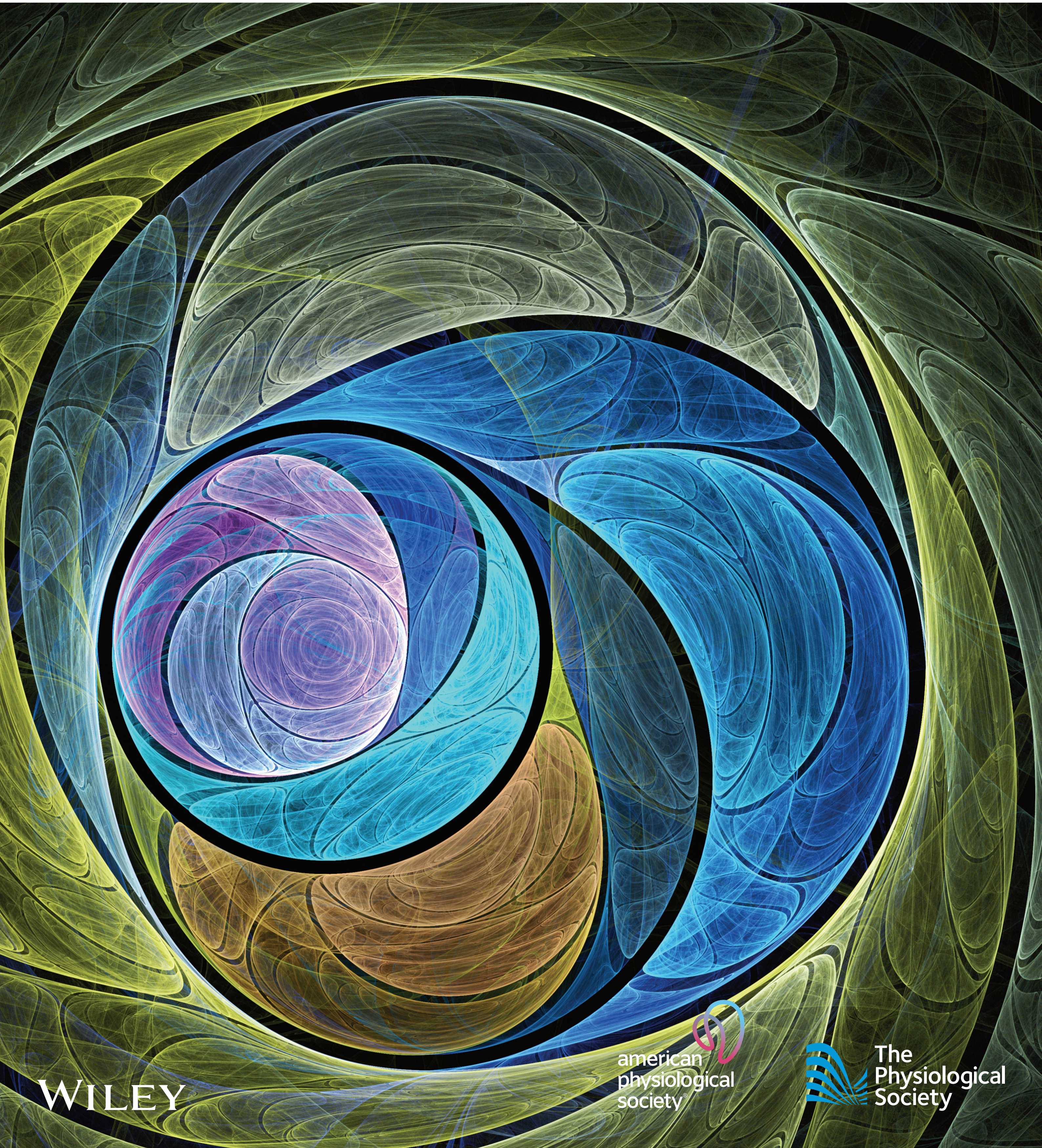

WILEY

american physiological society

The Physiological Society

Physiological Reports

Open Access

### Aims and Scope

*Physiological Reports* is an online only, open access journal that will publish peer-reviewed research across all areas of basic, translational and clinical physiology and allied disciplines. *Physiological Reports* is a collaboration between The Physiological Society and the American Physiological Society, and is therefore in a unique position to serve the international physiology community through quick time to publication while upholding a quality standard of sound research that constitutes a useful contribution to the field. Papers will be accepted solely on the basis of scientific rigor, adherence to technical and ethical standards, and evidence that the study is sufficiently well-conceived and the data support the conclusions.

*Physiological Reports* is interested in cellular and molecular studies as well as intact tissues, model organisms and translational studies. We welcome the work of biomedical scientists, whose studies incorporate physiological findings. Papers with negative data or confirmatory results are acceptable as long as they are well conceived. Papers concerned with modeling or new methods are acceptable as long as they are of physiological interest. For further information, see the journal's full scope statement available at http://onlinelibrary.wiley.com/journal/10.1002/(ISSN)2051-817X/homepage/ProductInformation.html

### Open Access and Copyright

All articles published by *Physiological Reports* are fully open access: immediately freely available to read, download and share. All *Physiological Reports* articles are published under the terms of the Creative Commons Attribution (CC BY) License, which permits use, distribution and reproduction in any medium, provided the original work is properly cited.

Copyright on any research article in a journal published by *Physiological Reports* is retained by the author(s). Authors grant Wiley a license to publish the article and identify itself as the original publisher. Authors also grant any third party the right to use the article freely as long as its integrity is maintained and its original authors, citation details and publisher are identified. Further information about the open access license and copyright can be found at http://www.wileyopenaccess.com/details/content/12f25db4c87/Copyright–License.html.

### Purchasing Print Reprints

As this is an open access journal, you have free, unlimited access to your article online. However, if you wish to obtain printed reprints, these may be ordered online: https://caesar.sheridan.com/reprints/redir.php?pub=10089&acro=PHY2

### Disclaimer

The Publisher and Editors cannot be held responsible for errors or any consequences arising from the use of information contained in this journal; the views and opinions expressed do not necessarily reflect those of the Publisher and Editors, neither does the publication of advertisements constitute any endorsements by the Publisher and Editors of the products advertised.

Wiley Open Access articles posted to repositories or websites are without warranty from Wiley of any kind, either express or implied, including, but not limited to, warranties of merchantability, fi tness for a particular purpose, or non-infringement. To the fullest extent permitted by law Wiley disclaims all liability for any loss or damage arising out of, or in connection with, the use of or inability to use the content.

Cover Image © Nataliia Yudina/Adobe Stock

### Editor-in-Chief

Josephine C. Adams
*University of Bristol, UK*

### Deputy Editor

David Wright
*University of British Colombia, Canada*

### Founding Editor

Susan Wray
*University of Liverpool, UK*

### Associate Editors

Oliver J. Price
*University of Leeds, UK*
Natasha Rogers
*Westmead Institute for Medical Research, Australia*
Sharon Rounds
*Brown University, USA*
Akiyuki Taruno
*Kyoto Prefectural University of Medicine, Japan*
Arianne L. Theiss
*University of Colorado Denver, USA*
Shizuka Uchida
*Aalborg University, Denmark*

### Consulting Editors

To be announced

### Social Media Coordinator:

Christine Klemens

### Editorial Board Members

Charlie Affourtit
*Plymouth University, UK*
B. Rita Alevriadou
*The State University of New York, USA*
Francisco Altamirano
*Houston Methodist Research Institute, Weill Cornell Medical College, USA*
Nadia Ameen
*Yale University, USA*
M. Safwan Badr
*Wayne State University, USA*
Ulf Baumgaertner
*Medical School Hamburg, Germany*
Brad Behnke
*Kansas State University, USA*
Andreas Beyer
*Medical College of Wisconsin, USA*
Vivek Bhalla
*Stanford University, USA*
Cary R. Boyd-Shiwarski
*University of Pittsburgh, USA*
Patrice Brassard
*Universite Laval, Canada*
Arthur J. Cheng
*York University, Canada*
Sam Cheng
*University of Florida, USA*
Gaurav Choudhary
*Brown University, USA*
Troy Cross
*University of Sydney, Australia*
Chunsun Dai
*Nanjing Medical University, China*
Glen Davison
*University of Kent, UK*
Katherine Dibb
*University of Manchester, UK*
Joanna Diong
*University of Sydney, Australia*
Lucy F. Donaldson
*University of Nottingham, UK*
Heather Drummond
*University of Mississippi, USA*
Carrie A. Duckworth
*University of Liverpool, UK*
Faadiel Essop
*Stellenbosch University, South Africa*
Stefanie Fenske
*LMU Munich, Germany*
Eric Gartman
*Brown University, USA*
Paige C. Geiger
*Kansas University Medical Center, USA*
Matthew Gorr
*The Ohio State University, USA*
Kirk Hamilton
*University of Otago, New Zealand*
Jules Hancox
*University of Bristol, UK*
Ilkka Heinonen
*University of Turku, Finland*
Connie C. W. Hsia
*University of Texas-Southwestern, USA*
Li Hu
*Chinese Academy of Sciences, China*
Andrew James
*University of Bristol, UK*
Matthew D. Jankowich
*Brown University, USA*
Thomas Andrew Jepps
*University of Copenhagen, Denmark*
Mirajul H. Kazi
*Catalent Pharma, Baltimore, USA*
Simon Keely
*University of Newcastle, Australia*
Brian Layden
*University of Illinois, USA*
Gareth Leng
*University of Edinburgh, UK*
David Levinthal
*University of Pittsburgh, USA*
Rebecca E. K. MacPherson
*Brock University, Canada*
Jennifer L. Maher
*University of Bath, UK*
Janice Marshall
*University of Birmingham, UK*
Chris McGlory
*McMaster University, Canada*
Jonathan Nizar
*University of Iowa Carver College of Medicine, USA*
Paul O'Connor
*Augusta University, USA*
Alan Pao, Stanford University, USA
*Jean-Francois Perrier*
University of Copenhagen, Denmark
Sandrine V. Pierre
*Marshall Institute for Interdisciplinary Research, USA*
Helle Praetorius
*Aarhus University, Denmark*
S. Russ Price
*East Carolina University Brody School of Medicine, USA*
Mrinalini C. Rao
*University of Illinois, USA*
Evan Ray
*University of Pittsburgh, USA*
Megan J. Ritter
*Boston University School of Medicine, Boston Medical Center, USA*
Alexander Rosenberg
*Midwestern University, USA*
Pedro Salas
*University of Miami, USA*
Ciara M. Shaver
*Vanderbilt University Medical Center, USA*
Bing Shen
*Anhui Medical University, China*
Larissa Shimoda
*John Hopkins University, USA*
Sunder Sim-Lucas
*University of Pittsburgh, USA*
Claire M. Smith
*University College London, UK*
Roderick Tan
*University of Pittsburgh, USA*
Christopher M. Waters
*University of Kentucky, USA*
Mathias Wernbom
*University of Gothenburg, Sweden*
Hongwei Yao
*Brown University, USA*
Hanry Yu
*National University of Singapore, Singapore*
Lili Zhou
*Southern Medical University, China*
